# Aneuploidy Enables Cross-Adaptation to Unrelated Drugs

**DOI:** 10.1093/molbev/msz104

**Published:** 2019-04-27

**Authors:** Feng Yang, Flora Teoh, Alrina Shin Min Tan, Yongbing Cao, Norman Pavelka, Judith Berman

**Affiliations:** 1 Department of Molecular Microbiology and Biotechnology, School of Molecular Cell Biology and Biotechnology, The George S. Wise Faculty of Life Sciences, Tel Aviv University, Tel Aviv, Israel; 2 Singapore Immunology Network (SIgN), Agency for Science, Technology and Research (A*STAR), Singapore; 3 Department of Vascular Disease, Shanghai TCM-Integrated Hospital, Shanghai University of Traditional Chinese Medicine, Shanghai, China; 4 Shanghai TCM-Integrated Institute of Vascular Disease, Shanghai, China

**Keywords:** evolution via aneuploidy, *Candida albicans*, antifungal responses, cross-adaptation, drug tolerance, drug resistance

## Abstract

Aneuploidy is common both in tumor cells responding to chemotherapeutic agents and in fungal cells adapting to antifungal drugs. Because aneuploidy simultaneously affects many genes, it has the potential to confer multiple phenotypes to the same cells. Here, we analyzed the mechanisms by which *Candida albicans*, the most prevalent human fungal pathogen, acquires the ability to survive both chemotherapeutic agents and antifungal drugs. Strikingly, adaptation to both types of drugs was accompanied by the acquisition of specific whole-chromosome aneuploidies, with some aneuploid karyotypes recovered independently and repeatedly from very different drug conditions. Specifically, strains selected for survival in hydroxyurea, an anticancer drug, acquired cross-adaptation to caspofungin, a first-line antifungal drug, and both acquired traits were attributable to trisomy of the same chromosome: loss of trisomy was accompanied by loss of adaptation to both drugs. Mechanistically, aneuploidy simultaneously altered the copy number of most genes on chromosome 2, yet survival in hydroxyurea or caspofungin required different genes and stress response pathways. Similarly, chromosome 5 monosomy conferred increased tolerance to both fluconazole and to caspofungin, antifungals with different mechanisms of action. Thus, the potential for cross-adaptation is not a feature of aneuploidy per se; rather, it is dependent on specific genes harbored on given aneuploid chromosomes. Furthermore, pre-exposure to hydroxyurea increased the frequency of appearance of caspofungin survivors, and hydroxyurea-adapted *C. albicans* cells were refractory to antifungal drug treatment in a mouse model of systemic candidiasis. This highlights the potential clinical consequences for the management of cancer chemotherapy patients at risk of fungal infections.

## Introduction

The increased risk of opportunistic infections is of particular concern for cancer patients undergoing chemotherapy, because of the immunosuppression that accompanies it (Teoh and Pavelka 2016). A leading cause of such infections is *Candida albicans*, a common member of the commensal microbiome (Brown et al. 2012; Mousset et al. 2014; Lai et al. 2019). Classical chemotherapeutic compounds target eukaryotic DNA synthesis and cell division and are often mutagenic (Ferguson and Pearson 1996). Core eukaryotic biochemical and cell biological processes, such as DNA synthesis and cell division, are highly conserved between humans and fungi, which limits the number of antifungal agents: only five classes of antifungals are available (Campoy and Adrio 2017), with only three of them in clinical use. Accordingly, chemotherapeutic drugs could have unintentional side effects on eukaryotic members of the host microbiota, including commensal fungi such as *C. albicans*.

Eukaryotic cells adapt to a broad range of stresses, with a classic example being the rapid adaptation of yeast cells to antifungal therapies (Cowen and Lindquist 2005; Bennett et al. 2014; Berman 2016). This is of great clinical relevance: The rapid development of resistance to fungistatic drugs such as fluconazole (FLC) is well documented for both *Candidaglabrata* and *C. albicans* (Borst et al. 2005; Berkow and Lockhart 2017). Importantly, resistance to the newer echinocandin class of fungicidal drugs, such as micafungin and caspofungin, also can appear within a few days of treatment in *C. albicans, C. glabrata*, and *Candidakrusei* (Pelletier et al. 2005; Baixench et al. 2007; Lewis et al. 2013; Forastiero et al. 2015; Sasso et al. 2017), as well as in *Candidaauris*, which is intrinsically FLC-resistant and has caused multidrug-resistant outbreaks across the globe (“*Candida auris* clinical update—September 2017,” 2017).

A well-document mechanism by which FLC resistance is rapidly acquired in *C. albicans* is via aneuploidy (Perepnikhatka et al. 1999; Selmecki et al. 2006; Rustchenko 2007; Selmecki et al. 2010; Brimacombe et al. 2018). Aneuploidy, defined as an imbalance in the number of whole chromosomes or chromosomal segments, arises at relatively high frequency in eukaryotic cells (Lee et al. 2010; Sterkers et al. 2012; Gallone et al. 2016; Gasch et al. 2016; Zhu et al. 2016). *C. albicans*, which does not undergo classical meiosis, has a high degree of genome plasticity and a high tolerance for aneuploidy (Rustchenko 2007; Selmecki et al. 2010). Aneuploidies often incur a fitness cost, reducing adaptation to many growth conditions (Torres et al. 2007; Sheltzer and Amon 2011). Yet, aneuploidy sometimes provides a selective advantage, with randomly acquired aneuploidies often able to confer beneficial properties under particular stress conditions (Pavelka et al. 2010). These characteristics are not unique to *C. albicans*: wild and clinical isolates of the model yeast *Saccharomycescerevisiae* often carry aneuploidies as well (Sunshine et al. 2015; Gallone et al. 2016; Gasch et al. 2016; Zhu et al. 2016; Peter et al. 2018). Furthermore, whole chromosome and segmental aneuploidies are often detected during in vitro evolution (Adams et al. 1992; Perepnikhatka et al. 1999; Koszul et al. 2004; Rancati et al. 2008; Gresham et al. 2010; Liu et al. 2015), and are common mechanisms of suppressing the deleterious effects of specific deletion mutations (Hughes et al. 2000; Rancati et al. 2008; Liu et al. 2015). In all cases where the molecular mechanism was determined, the adaptive value of a specific aneuploidy to a specific environment has been attributable to the altered copy number of one or more specific genes on the aneuploid chromosome (Rancati et al. 2008; Selmecki et al. 2008; Gresham et al. 2010; Pavelka, Rancati, and Li 2010; Liu et al. 2015; Sunshine et al. 2015).

Adaptation to one environment often affects fitness in an unrelated environment. For example, antagonistic pleiotropy causes inherent fitness tradeoffs between selected and unselected traits (Qian et al. 2012; Kessi-Perez et al. 2016). Alternatively, neutral accumulation of deleterious mutations in genes unnecessary in one selected environment could lead to fitness loss in another environment (Chun and Fay 2011; Hartfield and Otto 2011). But the fitness effects of adaptive mutations need not always be negative in unselected environments. In fact, experimental evolution of bacteria or yeast under one environmental condition sometimes leads to the acquisition of selective advantages in a second, unselected condition (Ferrari et al. 2009; Roux et al. 2015; Hampe et al. 2017). We refer to this phenomenon as “cross-adaptation.” Cross-adaptation can be explained by pleiotropic side effects of adaptive mutations (Travisano et al. 1995; Velicer 1999; Lázár et al. 2014) or by hitchhiking of unselected mutations due to genetic linkage with an adaptive mutation (Guttman and Dykhuizen 1994).

Because aneuploidy is associated with large and pleiotropic fitness effects across different environments (Pavelka et al. 2010), it raises the possibility that selection for aneuploidy of a particular chromosome in one environment could bias the adaptation of the organism to another environment (Chen et al. 2015; Sunshine et al. 2015). Despite the large number of genes affected by a single chromosomal aneuploidy, and the resulting potential of aneuploidy to drive a large number of adaptive changes, its role in cross-adaptation has received little attention. Most studies on adaptation have focused on infrequent and small genome changes, such as point mutations. Yet, large-scale genome changes, such as changes in chromosome number or structure, occur much more frequently and simultaneously affect larger numbers of genes, making them more likely to produce pleiotropic side effects (Storchova et al. 2006; Chen, Rubinstein, et al. 2012). Furthermore, the acquisition of aneuploidy may provide a transient, albeit unstable and imperfect, solution to a given stress condition that facilitates the acquisition of more beneficial and stable mutations in the long run (Yona et al. 2012).

Here, we address these gaps by testing the hypothesis that fungi adapt to chemotherapy using similar genetic mechanisms as those underlying adaptation to antifungal drugs, thus opening the door to potential cross-adaptation between the two classes of drugs. We posit that such cross-adaptation can, in turn, influence the progression and treatment of opportunistic infections, such as those caused by *C. albicans*. We find that adaptation of *C. albicans* to both chemotherapeutic and antifungal compounds is largely attributable to the acquisition of specific whole-chromosome aneuploidies and that genes on the aneuploid chromosome required for survival under hydroxyurea (HU) are not required for survival in caspofungin (CSP). In particular, we show that pre-exposure of *C. albicans* to the cancer chemotherapy drug HU potentiates tolerance to CSP, and that HU-adapted *C. albicans* isolates are refractory to CSP treatment in a mouse model of systemic candidiasis. Similar cross-adaptation was seen between echinocandin and azole classes of antifungals, which raise concerns about rapid mechanisms of adaptation to the two most widely used antifungal drugs. Thus, cross-adaptation may have important clinical implications: specific antifungal and chemotherapeutic agents may select for the adaptation of commensal fungi to unrelated antifungal therapies, thereby further limiting the number of antifungal drugs that can be used to treat these infections.

## Results

### Aneuploidy Is Associated with Rapid Adaptation to Anticancer Drugs

**Fig. 1.**
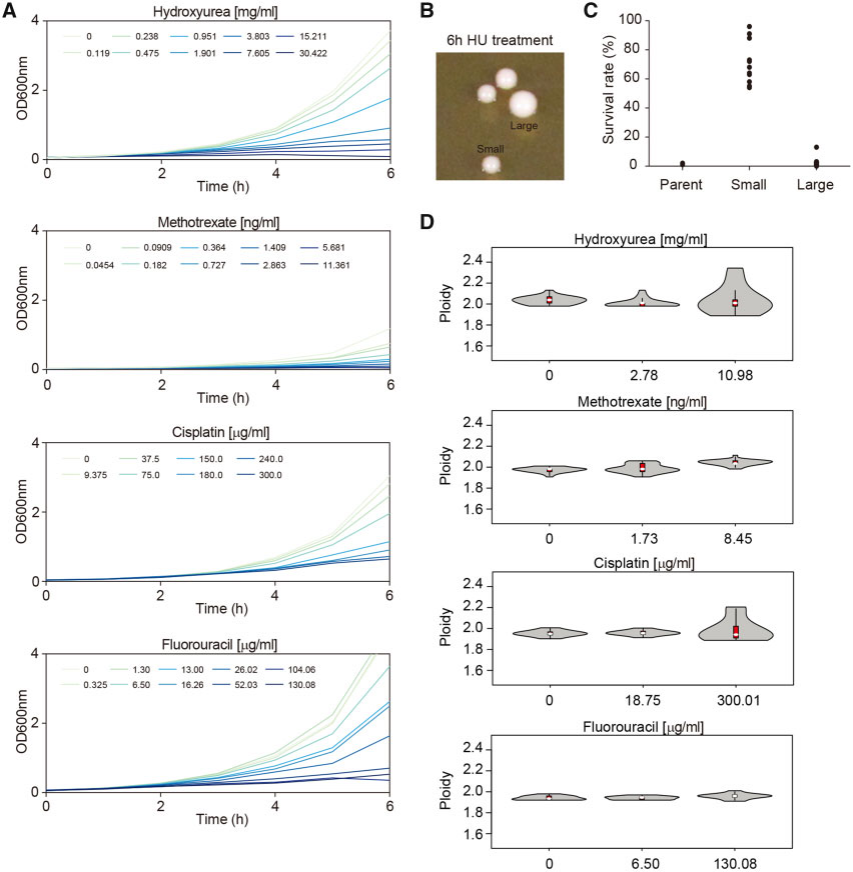
*Candida albicans* survivors of anticancer drugs show unstable colony phenotypes and variable ploidy levels. (*A*) Dose–response curves of *C. albicans* SC5314 exposed to a range of concentrations of the following anticancer drugs: hydroxyurea (HU), methotrexate (MTX), cisplatin (CDDP), and 5-fluorouracil (5-FU). Cultures were grown in YPD with HU, CDDP, or 5-FU, and in YNB with MTX. Data shown are the average of three technical repeats. Cell growth was monitored at intervals of 60 min in 100-ml flasks and 20 ml of culture medium at 37 °C for up to 6 h, with an initial cell density of 10^6^ cells/mL. OD readings were obtained using cuvettes and a spectrophotometer. As previously reported, HU induced filamentous growth, which skews OD readings upward, but this had no qualitative consequence on the conclusion that HU dose-dependently inhibits *C. albicans* growth. (*B*) Small and large colonies appearing on rich medium with no drug (YPD), after restreaking cells that originally survived 2.814 mg/ml HU treatment. (*C*) Ability of small and large colonies, obtained from (*B*), to survive (i.e., to form colonies) in HU. Survival rate was calculated as the percentage of colonies growing on 2.814 mg/ml HU relative to the total number of colonies growing on YPD medium without drug. (*D*) Range of ploidy levels, determined by flow cytometry analysis of DNA content, in cells that survived exposure to the drug at the indicated concentrations. The ploidy distributions are shown as violin plots. The interquartile range is represented by a red box, whereas the median value is indicated by a white dot.

To determine the susceptibility of *C. albicans* laboratory strain SC5314 to chemotherapeutic drugs, we measured its ability to grow in the presence of different antimitotic, antimetabolite, or DNA-damaging agents used for cancer chemotherapy. No changes in growth were detected with doxorubicin, paclitaxel, or cyclophosphamide, even at the highest concentrations used (supplementary fig. S1, Supplementary Material online). In contrast, when treated with HU, cisplatin, 5-fluorouracil (5-FU), or methotrexate (MTX), cells exhibited a dose-dependent growth inhibition (fig. 1*A*).

Cells that survived an acute exposure to HU displayed heterogeneity in colony sizes when plated on drug-free media (fig. 1*B*). Upon restreaking of small colonies, they gave rise to primarily small and a few large colonies. A striking phenotypic difference between the small and large colonies on drug-free plates became evident when they were replated to medium containing HU: although small colonies survived on HU plates, large colonies did not (fig. 1*C*). This suggests that adaptation to the drug in the small colonies was a reversible phenotype that was lost in the large colonies.

Because such unstable adaptation was reminiscent of aneuploidy (Yona et al. 2012), we assessed whole-cell genome content of a large number of strains, following 6 h of exposure to HU, cisplatin, 5-FU, or MTX. Cellular DNA content varied across several different drug treatments, and most especially at the highest concentrations tested, with exposure to HU yielding the highest frequency of altered DNA content (fig. 1*D*). Given the short time frame (6 h) after which these alterations were evident, this suggests that acute exposure of *C. albicans* to several chemotherapeutic compounds rapidly alters genome size, either by rapidly inducing the formation of aneuploid chromosomes (Harrison et al. 2014) or by strongly selecting for standing karyotypic variation (see Discussion).

To ask which chromosomes might be present in altered copy number, we determined the absolute copy number of each chromosome using a modified version of a previously described quantitative Polymerase chain reaction (PCR)-based method (see Supplementary Material online). We karyotyped 30 colonies from untreated cultures at 6 h after start of experiment and none of these colonies showed any aneuploidy. We then isolated 30 colonies each from cultures 6 h after treatment with drug concentrations able to inhibit *C. albicans* growth by either 50% (IC_50_) or by 90% (IC_90_). A variety of different karyotypes was observed across the treatments, and especially in the HU-treated cells (supplementary fig. S1, Supplementary Material online). Of note, one particular aneuploidy, trisomy of Chr2 (Chr2x3), was observed in three independent HU-treatment experiments (supplementary fig. S1*D*–*G*, Supplementary Material online). The apparent recurrence of this particular karyotype led us to hypothesize that Chr2 trisomy may confer a selective advantage for growth in HU.

### Aneuploidy Is Associated with Rapid Adaptation to Antifungal Drugs

**Fig. 2.**
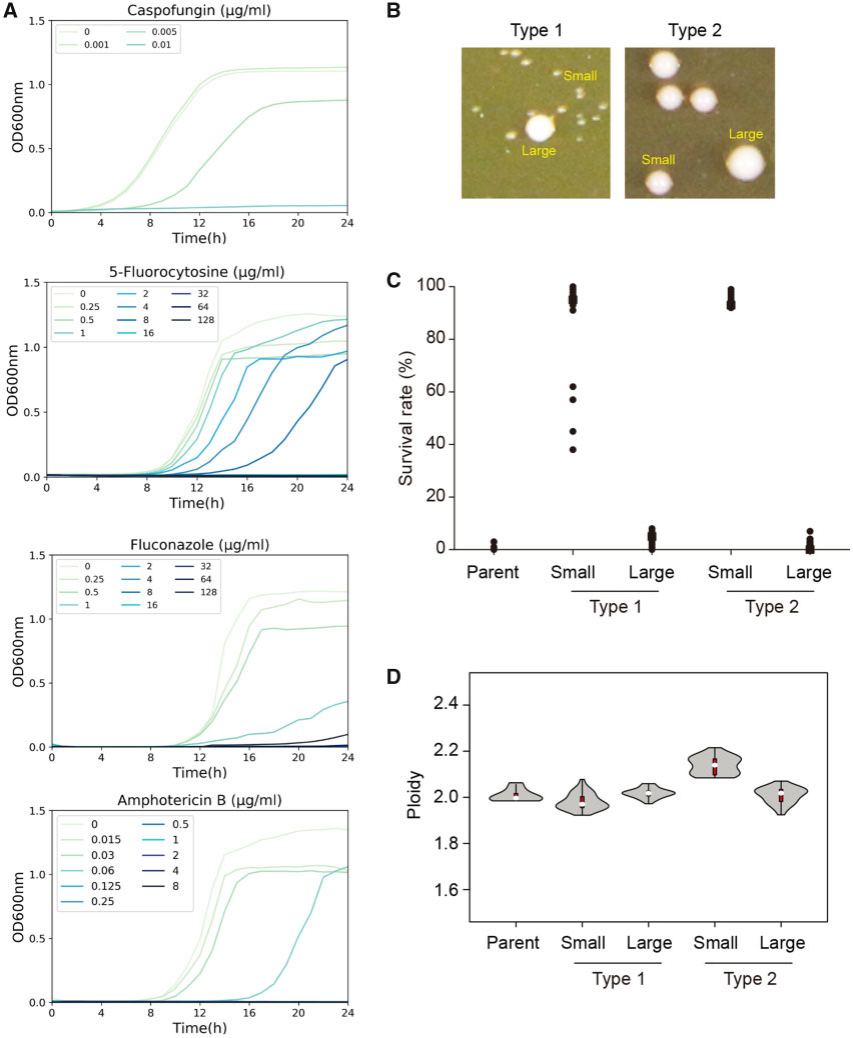
*Candida albicans* survivors of antifungal drugs show unstable colony phenotypes and variable ploidy levels. *(A)* Dose–response curves of *C. albicans* SC5314 exposed to caspofungin (CSP), 5-flucytosine (5-FC), fluconazole (FLC), and amphotericin B (AMB). Cell growth was monitored at intervals of 15 min in 96-well microtiter plates at 37 °C for 24 h. Data shown are the average of three technical repeats. *(B)* A range of colony sizes, primarily small with a few larger colonies, were evident when individual strains that survived 100 ng/ml CSP were replated onto rich medium with no drug (YPD). Based on the size difference between small and large colonies, two types of strains, as indicated, were distinguished. *(C)* Ability of small and large colonies, from (*B*), to survive (i.e., to form colonies) in 100 ng/ml CSP. Survival rate was determined as the percentage of colonies growing on 100 ng/ml CSP after 2 days of growth at 37 °C out of the total number of colonies that appeared on YPD medium without drug. (*n* = 32 and *n* = 28 for Type 1 and Type 2 strains, respectively.) *(D)* Range of ploidy levels, determined by flow cytometry analysis of DNA content, in cells that survived exposure to 100 ng/ml CSP. The ploidy distributions are shown as violin plots. The interquartile range is represented by a red box, whereas the median value is indicated by a white dot. (*n* = 8, 32 and 28, for the parental strain, Type 1 strains and Type 2 strains, respectively.)

In parallel experiments, we followed the growth of *C. albicans* SC5314 in a range of concentrations of different antifungal drugs, including those above the minimum inhibitory concentration (MIC) of each drug. As expected, dose-dependent growth inhibition was observed for CSP, 5-flucytosine (5-FC), FLC, and amphotericin B (AMB) (fig. 2*A*). Despite the lack of overall population growth, surviving colonies (i.e., survivors) did appear after 3 days or longer at supra-MIC CSP concentrations at a frequency of ∼10^−3^; and this result was consistent across all four major clades of *C. albicans* (supplementary fig. S1*I*, Supplementary Material online).

We plated ∼10^6^*C. albicans* SC5314 cells on Yeast extract–Peptone–Dextrose (YPD) plates containing 100 ng/ml CSP, incubated them for 3 days and observed ∼10^3^ survivor colonies. We then randomly picked 60 of these survivor colonies (20 large-, 20 medium-, and 20 small-sized colonies) and streaked each colony onto drug-free YPD plates. Strikingly, colonies on all 60 plates displayed colony size heterogeneity (fig. 2*B*). The majority (>98%) of colonies in drug-free media was smaller than parental colonies; larger colonies (with size similar to that of the parental colonies) appeared with low frequency. This suggested that the survivor state was unstable and biphasic. Among the independent survivors were two clearly distinguishable types of isolates: Type 1 survivors had a smaller average colony size on rich medium than the small Type 2 colonies (fig. 2*B*). In addition, Type 1 strains produced large colonies at lower frequency than Type 2 strains (0.1–0.5% vs. 1–2%). Interestingly, of the 20 survivors that produced a small colony on the original CSP plate, all displayed a Type 1 phenotype, whereas all 20 survivors that produced a large colony on the original CSP plate displayed a Type 2 phenotype; of the 20 medium-sized survivors, 14 were Type 1 and 6 were Type 2, instead. Regardless of the colony type, only the smaller, but not the larger, colonies derived from the original survivors retained the ability to survive in the presence of CSP (fig. 2*C*). This supports the idea that the larger colony derivatives were spontaneous revertants to the parental state.

This colony size instability of the survivors is a characteristic of the aneuploid state that has been reported for Chr5 monosomy (Chr5x1) isolates that survive in CSP (Yang et al. 2013). To test if the survival on CSP was due to Chr5x1, we asked if the survivors had undergone loss of heterozygosity at the mating type locus (*MTL)* on Chr5. Notably, all 32 analyzed Type 1 survivors (and none of 28 analyzed Type 2 survivors) were homozygous at *MTL* (supplementary fig. S2*A*, Supplementary Material online), consistent with possible loss of one copy of Chr5.

If survivors are aneuploid, we expect their whole-cell DNA content to differ from that of the parental strain. Analysis of Type 1 and Type 2 survivors by flow cytometry (Abbey et al. 2011) revealed that the small colony Type 1 survivors had lower DNA content than the wild-type diploid control (fig. 2*D*), consistent with a potential monosomy. By contrast, Type 2 survivor small colonies all exhibited ploidy levels significantly higher than the wild-type diploid control, suggesting possible extra copies of one or more chromosomes. The larger (parental-sized) colonies derived from both Type 1 and Type 2 survivors exhibited ploidy levels indistinguishable from the wild-type diploid control, consistent with the idea that these had reverted to the euploid state.

**Fig. 3.**
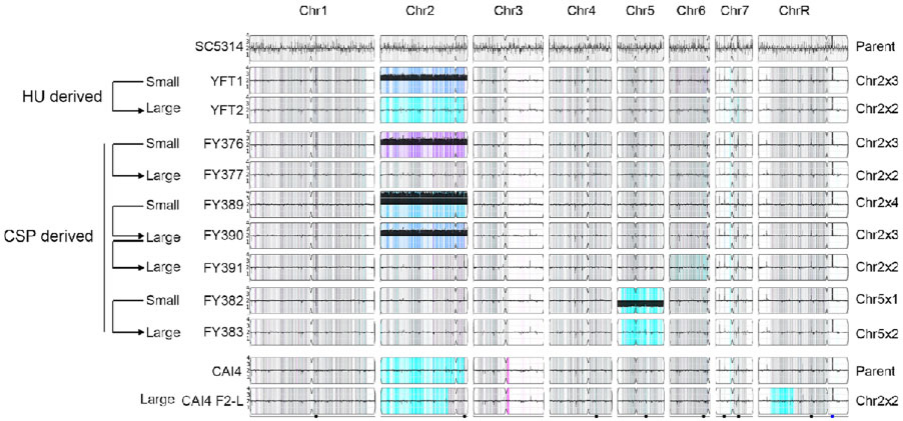
Y_MAP_ analysis of DNA content and allele ratios for HU and CSP survivor colonies reveals recurrent aneuploidies. Whole-genome DNA sequences for parents and the derived HU or CSP survivors were analyzed and displayed using Y_MAP_ (Abbey et al. 2014). Arrows on the left indicate the relationship between strains and the colonies derived from them. Relevant karyotype information is summarized to the right of each map. Read depth (normalized to that of the diploid parent) is shown on the *y*-axis on a log2 scale converted to absolute copy numbers (1–4). Allelic ratios (A:B) are color-coded: gray, 1:1 (A/B); cyan, 1:0 (A or A/A); magenta, 0:1 (B or B/B); purple, 1:2 (A/B/B); blue, 2:1 (A/A/B); light blue, 3:1 (A/A/A/B). The karyotype of strain CAI4 F2, which is euploid except for Chr2x3, was previously published and therefore not included in this analysis (Selmecki et al. 2005; Abbey et al. 2011).

Whole-genome sequencing of representative Type 1 and Type 2 small and large isolates was analyzed using Y_MAP_, which displays copy number and allelic frequency as a function of chromosomal position (Abbey et al. 2011; Hickman et al. 2013). The sequence revealed a monosomy of the entire chromosome 5 (Chr5x1) in a typical Type 1 small isolate (FY382), which reverted to the euploid state (Chr5x2) in a larger derived counterpart (FY383, fig. 3). Conversely, Type 2 small isolates (FY376) typically harbored an extra copy of Chr2 (Chr2x3), and their large colony derivatives (FY377) were euploid (Chr2x2). Interestingly, one particular Type 2 small isolate (FY389) had four copies of Chr2 (Chr2x4), which were sequentially lost in the FY390 (Chr2x3) and FY391 (Chr2x2) derivatives. Y_MAP_ analysis also confirmed that a HU survivor (YFT1), previously shown to carry increased DNA content (fig. 1*D*), indeed carried three copies of Chr2 (Chr2x3), and that one of its large colony derivatives (YFT2) was diploid (Chr2x2).

To test the generality of these findings, we used qPCR to karyotype all 60 original CSP survivors described in figure 2. All 60 strains carried one of three aneuploidies (supplementary fig. S2*B*, Supplementary Material online), that is Chr5x1 (32 strains), Chr2x3 (27 strains), or Chr2x4 (1 strain). Hence, these data indicate that aneuploidy was ubiquitous in the CSP survivors and was absent from the revertants that formed larger colonies on rich medium.

*GSC1* encodes the essential β-1, 3-glucan synthase subunit, which is the target of CSP (Douglas et al. 1997), and is known to harbor specific substitutions in CSP-resistant *C. albicans* strains (Park et al. 2005). To investigate the potential contribution of sequence alterations in this locus to the observed CSP adaptation mechanism, we sequenced two previously described *GSC1* mutational hot spots (Perlin 2007), but could not detect any nucleotide changes in either a Chr2x3 CSP survivor (FY376) or a Chr5x1 CSP survivor (FY382) (supplementary fig. S2*C*, Supplementary Material online). Although we cannot fully exclude the potential contribution of other types of mutations, taken together our data indicate that aneuploidy is a dominant mechanism of adaptation to CSP.

### Chr2 Trisomy Confers Adaptation to Both HU and CSP

**Fig. 4.**
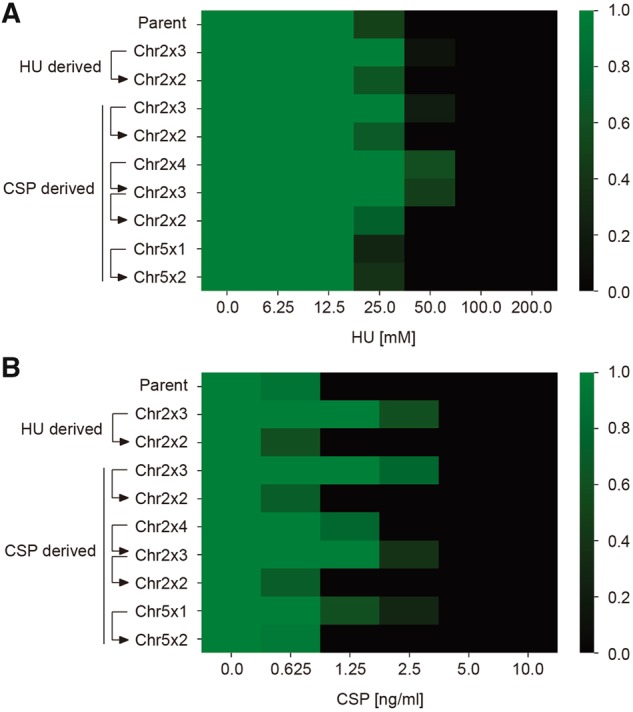
Extra copies of chromosome 2 increase growth ability in the presence of hydroxyurea and caspofungin. The growth ability of representative strains was tested in YPD medium supplemented with HU (*A*) or CSP (*B*) at concentrations indicated. Growth was measured by absorbance at 600 nm after 36 h at 37 °C. Optical densities were standardized to those of drug-free controls and averaged across three technical replicates. Data were quantitatively displayed as color intensities as indicated in the color bar key on the right, using a custom python script.

Based upon the above genome analyses, exposure to either HU or CSP yielded isolates with a convergent karyotype, that is, trisomy of Chr2 (Chr2x3). To test the hypothesis that Chr2x3 confers a selective advantage under both HU stress and CSP stress, we used the same isolates previously subjected to genome sequencing, and either cultured fixed amounts of cells in liquid media containing serial dilutions of the drug (fig. 4) or performed spot assays with serially diluted cells on plates containing fixed concentrations of drug (supplementary fig. S2*D*, Supplementary Material online). Both experiments revealed that, regardless of whether the Chr2x3 karyotype was acquired in response to selection on HU or on CSP, all Chr2x3 isolates exhibited improved growth under both HU and CSP relative to their euploid counterparts. Conversely, Chr5x1 conferred adaptation to CSP as well as increased tolerance to FLC (supplementary fig. S2*E* and *F*, Supplementary Material online), but did not improve growth in HU.

To exclude potential artifacts due to the prior exposure to a cytotoxic or fungicidal drug, we took advantage of a pair of strains that had never been exposed to either HU or CSP and that differed only by Chr2 copy number. CAI-4 is a classic *C. albicans ura3/ura3* auxotrophic derivative of lab strain SC5314 (Fonzi and Irwin 1993), and one of its derivative strains (F2) was previously found to be trisomic for Chr2 (Selmecki et al. 2005; Abbey et al. 2011). By screening several derivatives of F2, we isolated a strain (F2-L) that formed larger colonies on YPD plates due to spontaneous reversion to the euploid state (fig. 3), and we compared the ability of these two CAI-4 derivatives to grow in both HU and CSP. Consistent with the idea that an extra copy of chromosome 2 is sufficient to confer adaptation to both HU and CSP, only the F2 (Chr2x3), but neither the euploid parental CAI-4 nor the euploid derived F2-L (Chr2x2) strain, was adapted for growth on both drugs (supplementary fig. S2*D*, Supplementary Material online).

### *RNR1* and *RNR21* Contribute to HU Adaptation but Do Not Affect CSP Adaptation

**Fig. 5.**
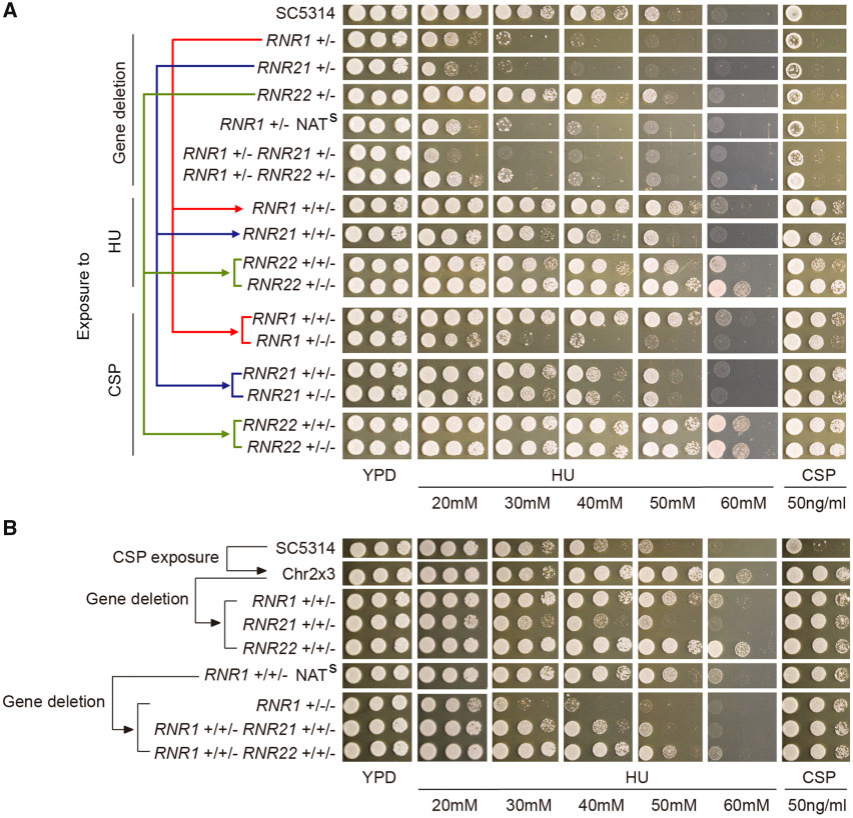
Role of RNR gene copy numbers for ability to grow in HU, but not in CSP. Strains with different numbers of alleles of *RNR1, RNR21*, and *RNR22* were constructed by one of two strategies: (*A*) by deletion of one gene copy from diploid strain SC5314, followed by chromosome duplication via exposure to HU or CSP or (*B*) by deletion of one gene copy from a Chr2 trisomic strain. Strains were spotted (3 µl/spot, 10-fold dilutions) onto HU or CSP plates and incubated at 37 °C for 48 h. All strains in (*A*) and in (*B*) were analyzed on the same drug plates. Comparison of mutant growth should be made between euploid strains or between trisomic strains. For example, the Chr2x3 strain (FY376) is a control for all strains in (*A*) and (*B*).

Given that Chr2x3 appeared to be both necessary and sufficient for adaptation to two stresses, that is HU and CSP treatment, we hypothesized that the presence of extra copies of one or more genes on Chr2 could provide the molecular basis accounting for these phenotypes. HU inhibits ribonucleotide reductase (RNR) activity, and three out of four RNR genes are present on Chr2 (*RNR1*, *RNR21*, *RNR22*). Thus, we tested the contribution of these *RNR* genes to growth in HU or CSP. In the diploid SC5314 background, the *RNR1*/*rnr1* and *RNR21*/*rnr21* heterozygous deletion strains grew very poorly in the presence of HU, whereas the growth of the *RNR22*/*rnr22* heterozygous knockout strain was indistinguishable from the parental SC5314 strain (fig. 5*A*). Next, we deleted one copy of each *RNR* gene in the Chr2x3 background (FY376); the loss of one copy of either *RNR1* or *RNR21* was sufficient to eliminate HU resistance in this Chr2x3 strain, whereas the loss of one copy of *RNR22* did not have any obvious effect on the HU response (fig. 5*B*). We also used a complementary strategy (based on CSP or HU exposure of heterozygous deletion euploid strains) to generate Chr2x3 strains that lacked one or two copies of *RNR1*, *RNR21*, or *RNR22* while retaining three copies of the other two *RNR* genes (supplementary fig. S2*G*, Supplementary Material online). In general, extra copies of *RNR1* and *RNR21* improved growth, with extra *RNR21* having a more dramatic effect; *RNR22* had no detectable effect on growth in HU. Overall, these results support the idea that Chr2x3 mediates HU resistance by simultaneously increasing the number of copies of *RNR1* and *RNR21*.

Notably, all Chr2x3 strains grew better than euploid strains on CSP, regardless of RNR gene deletion status (fig. 5), indicating that RNR genes play no role in CSP survival. Thus, although Chr2x3 increases growth on both HU and CSP, the genes responsible for growth on HU are not the same as those responsible for survival in CSP. Consistent with this, although selection of HU survivors from diploid heterozygous *RNR1/rnr1* or *RNR21/rnr21* strains yielded Chr2x3 strains where the duplicated Chr2 homolog was exclusively the one carrying the intact copy of the *RNR* gene, selection of CSP survivors from the same starting strains yielded Chr2x3 survivors where the Chr2 duplication could occur on either homolog (supplementary fig. S2*G*, Supplementary Material online).

### The Calcineurin Pathway Does Not Contribute to Survival in HU

**Fig. 6.**
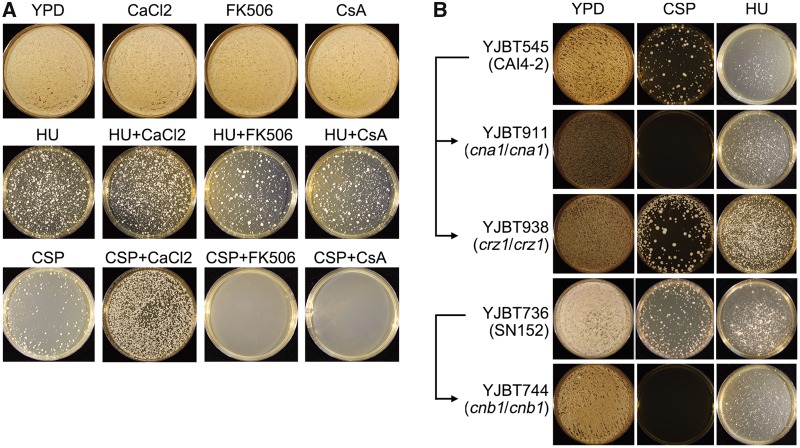
Calcium and the calcineurin pathway are required for improved survival on caspofungin, but not on hydroxyurea. (*A*) Parental strain SC5314 was plated (∼10^6^ cells per plate) onto YPD supplemented with HU (2.814mg/ml) or CSP (100 ng/ml) alone, or in combination with calcium chloride (100 mM) or inhibitors of the calcineurin pathway, FK506 (0.5 µg/ml) and cyclosporin A (CsA) (0.5 µg/ml), as indicated. (*B*) Parental strains YJBT545 and YJBT736, and isogenic derivatives lacking genes *CNA1, CNB1* (encoding calcineurin subunits) or *CRZ1* (encoding a transcription factor downstream of the calcineurin pathway) were plated on YPD plates supplemented with HU (2.814mg/ml) or CSP (100 ng/ml) at 37 °C for 3 days. Note that growth on CSP, but not HU, is enhanced with added calcium and inhibited both by inhibitors of, and mutants affecting, the calcineurin pathway.

The calcineurin pathway contributes to the ability of *C. albicans* to tolerate CSP (Walker et al. 2008). The calcineurin-responsive zinc finger 1 (Crz1) is a calcineurin-regulated transcription factor involved in azole tolerance (Onyewu et al. 2004) but calcineurin-mediated CSP tolerance was shown to be independent of Crz1 (Chen et al. 2014). To ask if the calcineurin pathway plays any role also in the HU adaptation mechanism, we tested the effect of calcium chloride (which enhances calcineurin activity), tacrolimus (FK506), or cyclosporin A (CsA)—two inhibitors of calcineurin activity with different binding mechanisms—on the ability to survive CSP or HU exposure (fig. 6*A*). As expected, survival on CSP was enhanced by calcium chloride (CaCl_2_) and inhibited by both calcineurin inhibitors (fig. 6*A*). Moreover, CSP survival was blocked by genetic deletion of either the catalytic (CnA) or regulatory (CnB) subunits of calcineurin, and unaffected by the removal of Crz1 (fig. 6*B*). In contrast, CaCl_2_, calcineurin inhibitors and mutations in either *CNA* or *CNB* showed little or no effect on the appearance of HU survivors (fig. 6). Taken together, these data show that, contrary to survival in CSP, adaptation to HU does not require the calcineurin pathway.

### Adaptation of *C. albicans* to HU Facilitates Cross-Adaptation to CSP

**Fig. 7.**
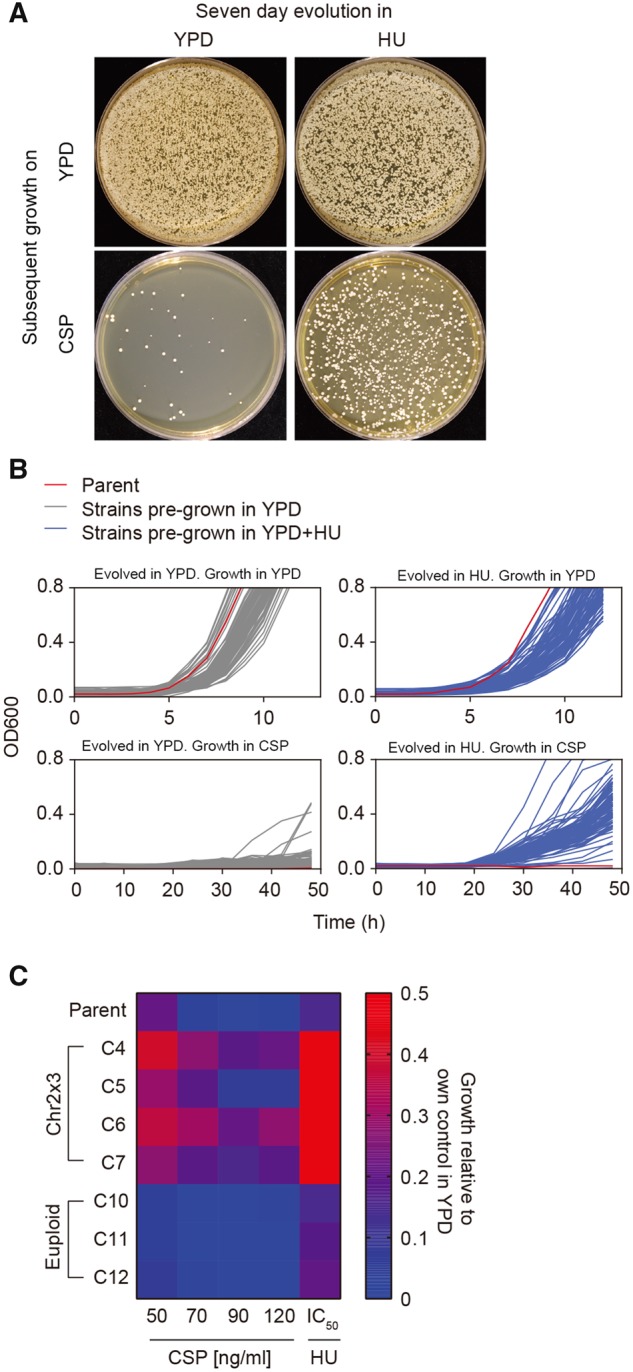
Effect of HU pre-exposure on cross-adaptation to caspofungin. Parental strain SC5314 was passaged daily for 7 days in YPD+HU (IC_50_; 2.814 mg/ml) at 37 °C, washed, and growth was measured on either solid (*A*) or liquid (*B*) medium. (*A*) ∼1 × 10^5^ cells were plated on YPD plates (with or without CSP [100 ng/ml] as indicated) and incubated at 37 °C for 3 days. (*B*) Passaged cells (with or without HU from the pre-exposure period above) were plated on YPD at a cell density of ∼200 CFUs/plate and incubated at 37 °C for 24 h. From each plate, 94 colonies from the control YPD treatment (gray curves), and 94 colonies from the YPD+HU treatment (blue curves) were randomly selected and grown at 37 °C in YPD with or without CSP (100 ng/ml); parental strain without pre-exposure was also tested (red curves). Growth was measured as absorbance at 600 nm every 15 min. (*C*) Strains evolved for 7 days as above were analyzed by spot assays. Serial dilutions of cell suspension were spotted (3 µl/spot) on YPD plates supplemented with the indicated concentrations of HU or CSP and incubated at 37 °C for 48 h. Each condition was tested a minimum of three independent times. Growth is represented in a heat map comparing strains with or without Chr2 trisomy (Chr2x3, euploid, respectively), as described in methods.

Aneuploidy often causes reduced fitness under some conditions (Thorburn et al. 2013) and thus we asked how exposure to HU (mimicking chemotherapy in the host) would affect the ability of *C. albicans* to adapt to CSP (as an antifungal therapy): would Chr2x3 isolates be fit enough to survive and provide a selective advantage under the second drug treatment? To address this question, we performed a short-term adaptive evolution experiment, in which we continuously exposed *C. albicans*, over 7 days of daily serial passaging, to a concentration of HU causing 50% growth inhibition in wild-type SC5314 cells (IC_50_). The cultures were then plated on either HU or CSP plates and the frequency of colony formation was monitored 3 days postincubation. Indeed, the number of colonies appearing on CSP plates was ∼10-fold higher for cultures pre-exposed to HU, relative to those passaged in parallel without drug exposure (fig. 7*A*). Cultures evolved in (i.e., adapted to) HU produced primarily colonies that were cross-adapted to CSP, and that grew with a slightly slower doubling time than the parent in the absence of drug (fig. 7*B*). To ask whether the CSP cross-adaptation was associated with Chr2x3, we randomly selected individual strains that had survived the 7-day HU evolution experiment, quantified their growth in spot assays and also analyzed their karyotypes (fig. 7*C*). Notably, HU-adapted isolates that were euploid had no effect on growth in CSP, whereas isolates that carried Chr2x3 generally showed improved fitness relative to the parent in CSP. The improved growth was more evident at lower CSP concentrations, with some differences between isolates. Thus, prior exposure and adaptation to HU increases the frequency of survival on CSP, presumably via the increased prevalence of Chr2x3 in the population of HU survivors. Consistent with this hypothesis, 9 of 12 colonies isolated after an independent 7-day evolution experiment in HU IC_50_ carried a trisomy of chromosome 2 (data not shown). This also implies that cancer chemotherapy with HU has the potential to promote cross-adaptation to CSP.

### Relevance *In Vivo*

**Fig. 8.**
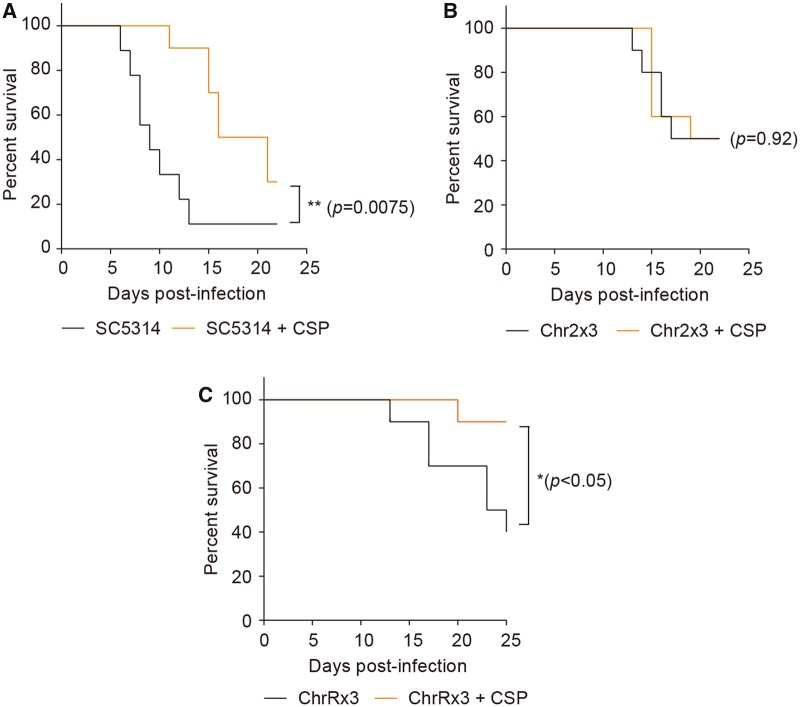
Survival curves of C57BL/6J mice systemically infected with wild-type or HU-evolved *Candida albicans* strains harboring either ChrRx3 or Chr2x3, with or without CSP treatment. CSP was administered intraperitoneally at a dose of 0.063 mg/kg body weight daily for 7 days, beginning 1 day after infection. Control mice were administered 100 μl of phosphate buffered saline intraperitoneally. Survival curves were compared using the log-rank test.

CSP is a first-line antifungal drug used in clinics for the treatment of systemic candidiasis (Pappas et al. 2016). The observation that aneuploidy can be generated by chemotherapeutic treatment (fig. 1), and that Chr2x3, whether selected during exposure to HU or CSP, promotes survival in either of the drugs (fig. 4), suggests that HU treatment of cancer patients has the potential to select for commensal *C. albicans* isolates that would be more recalcitrant to CSP antifungal therapy. To begin to address this issue and determine if it has the potential to be clinically relevant, we tested the response to CSP treatment in a mouse model of systemic candidiasis. As expected, CSP treatment during systemic infection with the diploid SC5314 significantly prolonged survival and also improved morbidity, as measured by weight loss (fig. 8*A* and supplementary fig. S3*A*, Supplementary Material online).

We next repeated these mouse infection experiments with a Chr2x3 strain isolated after 14 days of daily serial passaging (adaptive evolution) in the presence of HU, and which was highly adapted for growth in HU (supplementary fig. S3*D*, Supplementary Material online). In this case, mice showed neither a significant improvement in survival (fig. 8*B*), nor in morbidity (supplementary fig. S3*B*, Supplementary Material online), when they were treated with CSP. To control for possible general effects of aneuploidy and other changes induced by HU, we performed parallel infections with another HU-adapted aneuploid strain recovered after the same 14-day HU evolution experiment, this time bearing the ChrRx3 (supplementary fig. S3*D*, Supplementary Material online), instead of the Chr2x3, karyotype. We reasoned that trisomy R would cause a similar genetic burden as trisomy 2, as their chromosome sizes are similar. Although there was no significant improvement in morbidity (supplementary fig. S3*C*, Supplementary Material online), CSP treatment significantly improved survival during systemic infection with this ChrRx3 strain, as was the case of infection with SC5314 (fig. 8*C*). These results indicate that Chr2x3 is sufficient to eliminate the benefit of CSP *in vivo* during systemic infection, consistent with our observations of its increased CSP survival *in vitro*. Thus, aneuploidy generated by a chemotherapeutic drug can have profound effects on the treatment of systemic candidiasis, a common infection in cancer patients.

## Discussion

Antimicrobial drug resistance is a serious threat to the public (O'Neill 2016). Stewardship programs regulating the use of antibiotics aim to preserve our arsenal of anti-infective agents for future generations, based on the premise that excessive use of a particular antimicrobial agent will eventually select for reduced susceptibility to that same agent. The evolutionary mechanism behind this is obvious: antimicrobials impose strong selective pressures on pathogens, which eventually adapt to these treatments via positive selection of resistance mutations. A relatively underappreciated phenomenon is the emergence of resistance (or reduced susceptibility) to antimicrobial drugs due to nonselective evolutionary mechanisms, such as cross-adaptation. In some cases, the cross-adaptation mechanism is trivial and predictable. A classic example is the selection of β-lactamase-positive bacteria by one β-lactam compound conferring resistance to another, unselected, β-lactam drug—or the up-regulation of drug efflux pumps, which simultaneously confer resistance to multiple drugs in both bacteria and fungi. Cross-adaptation via acquisition of aneuploidy is unique in that the molecular mechanisms of adaptation to the two drugs (the selected and the unselected drug) can potentially be completely independent of each other because of the number of genes affected by the chromosome copy number change. This was evident here for trisomy of chromosome 2, which conferred adaptation to both HU and CSP, albeit by two different mechanisms: increased growth on HU (but not on CSP) was mediated by increased copy numbers of *RNR1* and *RNR21* genes; and increased survival on CSP (but not on HU) was mediated by a Crz1-independent calcineurin-mediated pathway.

The Chr2x3-mediated cross-adaptation between CSP and HU likely occurred simply because alleles underlying both adaptations were located on the same chromosome. Two major differences distinguish this type of hitchhiking from classic genetic linkage between two closely located resistance-causing mutant alleles. First, resistance alleles encoded on an aneuploid chromosome need not carry any sequence mutation, as they exert their adaptive effect via altered gene dosage as opposed to altered protein function. Consistent with this idea, aneuploid yeast strains differing solely by their chromosome copy number configurations, yet otherwise carrying identical genome sequences, display clearly distinguishable phenotypes including resistance to antifungal or chemotherapeutic drugs (Pavelka, Rancati, Zhu, et al. 2010), as well as a competitive advantage in *in vitro* competition experiments (Venkataram et al. 2016), in environmental isolates (Hose et al. 2015; Leducq et al. 2016), industrial yeasts (Gorter de Vries et al. 2017), and clinical isolates (Zhu et al. 2016). Second, alleles underlying aneuploidy-driven cross-adaptation, when encoded on the same chromosome, can be physically very distant from each other, as eukaryotic cells can acquire whole-chromosome aneuploidy via nonmeiotic, and hence nonrecombinogenic, processes such as chromosome nondisjunction. Accordingly, aneuploidy-mediated cross-adaptation has the potential to occur more frequently than previously appreciated. Consistent with this conclusion, high-throughput phenotypic profiling revealed several aneuploid *S. cerevisiae* strains displaying fitness advantages over their euploid counterparts in more than one condition, including growth on antifungals or chemotherapeutic drugs (Pavelka, Rancati, Zhu, et al. 2010). If confirmed, this conclusion would have important implications for antimicrobial stewardship programs, which would have to look beyond antimicrobials to other drugs that have the potential to select for cross-adaptation to those antimicrobials. This issue is highlighted by results with the mouse model of systemic candidiasis used here, where prior adaptation of *C. albicans* to the chemotherapeutic drug HU via acquisition of Chr2x3 aneuploidy impaired the therapeutic effect of the first-line antifungal CSP. If these data were translated to humans, they would suggest that cancer patients undergoing HU chemotherapy might be selecting for *C. albicans* strains cross-adapted to CSP—and if those HU-adapted strains were then to infect the patient, the infection would be more recalcitrant to CSP treatment.

Resistance to CSP is largely conferred by mutations within the “hot spot” regions of genes encoding beta-glucan synthase (Perlin 2015), however strains that do not carry these mutations and grow slowly in CSP are often isolated from patients (Castanheira et al. 2010; Shields et al. 2015). It is important to note that Chr2x3 and Chr5x1 aneuploids retained susceptible MIC (performed according to the Clinical & Laboratory Standards Institute (CLSI) standards) levels, yet continued to grow, albeit slowly, when replated onto 100 ng/ml CSP, a concentration ∼4-fold higher than the parental MIC (23 ng/ml). Thus, the ability of some cells to survive and divide, albeit slowly, in supra-MIC concentrations of CSP, is likely to be missed in conventional assays of susceptibility. This highlights that, despite the generally fungicidal nature of CSP in *C. albicans*, rare aneuploid strains in the population might not be killed by the drug and might continue to divide. Similarly, growth of Chr2x3 strains on HU was slower than growth in the absence of any drug. Importantly, during the course of an infection, the ability to survive and grow, even slowly, in either HU or CSP, has the potential to facilitate the acquisition of additional adaptive mutations in the pathogen and selective pressure would then promote adaptation of those individuals with improved fitness in the context of the host.

An open question arising from this study is related to the source of the adaptive aneuploidy. Is the drug merely selecting on standing karyotypic variation or inducing aneuploidy *de novo*? Mitotic errors leading to aneuploidy occur relatively frequently in fungi, even under optimal laboratory conditions (Zhu et al. 2014), leading to a large variety of karyotypes in both natural and clinical isolates (Hirakawa et al. 2015; Hose et al. 2015). Hence, standing karyotypic variation may be high in fungal populations, and thus not limiting to the selection processes described here. Moreover, most chemotherapeutic drugs, like HU, are mutagenic in nature and can therefore potentially increase the genetic variability in a population. Accordingly, a variety of different karyotypes were recorded as early as 6 h posttreatment with HU (used at its IC_50_), a time point that would not allow for enough cell divisions to select such a great diversity of karyotypes purely from standing variation. More recently, the antifungal agent FLC was shown to induce abnormal mitoses and to increase the probability of tetraploidy and aneuploidy in several yeast species (Harrison et al. 2014; Altamirano et al. 2017). Another possibility is represented by stress-induced aneuploidy, which occurs in both *C. albicans* (Forche et al. 2011) and *S. cerevisiae* (Chen, Bradford, et al. 2012). Since antifungal treatment is inherently stressful to the target cells and often induces stress responses, it is tempting to speculate that, like FLC, CSP may increase the proportion of aneuploid cells in a fungal population.

## Materials and Methods

### Strains and Growth Conditions

Strains used in this study are listed in supplementary table S1 (Supplementary Material online). Stock cultures of all strains were preserved in 35% glycerol and maintained at −80 °C. Unless otherwise specified, cells were grown in YPD media (1% [w/v] yeast extract, 2% [w/v] peptone, and 2% [w/v] d-glucose) at 37 °C in a shaking incubator at 150–200 rpm. For experiments involving solid media, 1.5% (w/v) agar was added to the plates, which were incubated without shaking. For the selection of gene knockout strains, YPD agar containing 300 μg/ml nourseothricin (Werner BioAgents) medium was used (YPD+NAT). To evict the disruption cassette, yeast nitrogen base (YNB)–bovine serum albumin (BSA) (0.17% [w/v] YNB, 2% [w/vw/v] d-glucose, 0.02% [w/v] BSA, 2% [w/v] agar) plates were used.

### Overnight Cultures

Cell were grown overnight in liquid medium for 15–16 h. Cultures were then relaunched into fresh medium and incubated for additional 2 h at 37 °C. Cells were harvested using a volume that would give a final optical density of 600 nm (OD_600_) of 0.03–0.05 in 20 ml YPD medium. Cells were collected at 13,000 rpm for 1 min and the pellet was resuspended in 20 ml of YPD either with or without the relevant drug as indicated. In experiments involving MTX, liquid YNB medium without amino acids (Becton-Dickinson) was used.

### Standard Growth Assays

Overnight cultures were prepared and relaunched in 20 ml medium as described above. Cell density was measured as OD_600_ readings taken hourly for at least 6 h. These readings were then log-transformed and plotted over time to obtain the growth chart. Growth rates were calculated as the slopes of linear regression curves fitted on the log-transformed OD_600_ readings over time.

### Acute Exposure to Chemotherapeutic Drugs

An overnight culture of SC5314 was diluted into fresh medium as above, aliquoted to a final OD_600_ of 0.03 − 0.05 in 20 ml of YPD, and then collected at 13,000 rpm for 1 min. Cell pellets were resuspended in 20 ml of liquid YPD medium either without chemotherapeutic drugs (control), or containing an amount of drug equivalent to the IC_50_ or IC_90_ of that drug as indicated. In cases where calculation of the IC_50_ and IC_90_ was not possible (as with cisplatin), the highest concentration for determining the dose–response relationship was used in lieu of the IC_90_. Growth rates were measured as in the dose–response experiment to ensure that growth inhibition by the drugs was within the expected ranges. At 6 h after treatment with a chemotherapeutic drug, cells were counted with a hemocytometer and then diluted with PBS to give a cell density of 2,000 cells/ml. Hundred microliters of this suspension was then spread on YPD agar without any chemotherapeutic drug to obtain single colonies. The plates were incubated overnight at 37 °C and then scanned using a desktop scanner. The images were processed by a custom Fiji/ImageJ plugin (Schindelin et al. 2012; Schneider et al. 2012). After this, a previously described R script was used to count colony numbers and measure colony size (Liu et al. 2015). Colonies were then binned according to size, and one representative colony from each bin (for a total of 12 colonies) was randomly selected for further analysis. These colonies were reinoculated into YPD in a deep 96-well block to obtain cells for flow cytometric analysis of ploidy (described below) and karyotyping by quantitative PCR (see Supplementary Material online).

### Ploidy Analysis

Ploidy was analyzed using flow cytometry as described previously with some modifications (Hickman et al. 2015). Strains were grown on YPD plates and several colonies were picked as described above, resuspended in YPD and incubated for 3 h at 37 °C. The cells were then harvested and washed twice with 50:50 TE, that is 50 mM ethylenediaminetetraacetic acid in 50 mM Tris (pH 8.0). Twenty microliters of cell suspension were fixed with 180 µl of 95% ethanol overnight at −20 °C, washed once with 50:50 TE and 100 µl of 1 mg/ml RNAse A (Macherey-Nagel) was added. Cells were then incubated at 37 °C for 1 h, washed once with 50:50 TE and resuspended in 50 µl of 5 mg/ml proteinase K (Promega). Cells were then incubated at 37 °C for 30 min, washed once with 50:50 TE and resuspended in 200 µl of Sybr Green I (Lumiprobe), which was a 1:67 dilution in 50:50 TE of the commercial stock, and incubated overnight in the dark. Cells were finally sonicated and analyzed in 96-well batches on a MACSQuant VYB instrument (Miltenyi Biotec). Data were analyzed using FlowJo software (version 10.4).

### Construction of RNR Gene Deletion Strains

*NAT1* flipper gene deletion cassette was amplified from plasmid pJK863 (Shen et al. 2005). Approximately 500 bp upstream region of the gene to be deleted was amplified from the genomic DNA of SC5314, such that its 3′ end overlapped with the 5′ end of the *NAT1* flipper; similarly, 500 bp downstream region of the target gene was amplified such that its 5′ end overlapped with the 3′ end of the *NAT1* flipper. The upstream region of each gene was then fused by PCR to the 5′ region of the cassette and the downstream region of the gene was fused by PCR to the 3′ region of the cassette, such that the two PCR products had an overlap of 500–1,000 bp within the cassette. All primer sequences used for plasmid and strain construction are listed in supplementary table S2 (Supplementary Material online). The upstream and downstream fusion products (split markers) for each gene were then simultaneously transformed in *C. albicans* following the lithium acetate method (Wilson et al. 2000). Transformants were selected on YPD+NAT agar plates. The replacement of the gene with the *NAT1* flipper cassette was confirmed by diagnostic PCR, using primers that annealed outside the flanking homology regions. The *NAT1* flipper was then evicted by streaking the clones on YNB-BSA plates, which induces the Flp recombinase.

### Generation of Isogenic HU-Induced Aneuploid and Euploid Strains

Three trisomic Chr2 strains were selected from short-term growth assays, two of which were derived from HU IC_50_ treatment, and one from IC_90_ treatment. These strains were passaged twice on YPD agar in the absence of HU. Prospective colonies were selected on the basis of colony size (as aneuploid strains tend to grow slower than their euploid counterparts in the absence of relevant selective forces). After the second passage, single colonies were karyotyped using flow cytometry and qPCR (as detailed above).

### Adaptive Evolution Experiments in *C. albicans*

SC5314 was cultured overnight at 37 °C in a shaker (150 rpm). Cells were then diluted to an OD_600_ of 0.01 in 20 ml YPD, containing 2.814 mg/ml (IC_50_) of HU. Cultures were passaged daily by diluting cells to an OD_600_ of 0.01 into fresh YPD containing the same concentration of HU. At each passage, cultures were diluted in PBS and spread on YPD agar to obtain single colonies for subsequent analysis of ploidy and karyotyping, as well as to assess colony size variation of the population using a custom R script.

### Effect of CSP Treatment during Systemic *C. albicans* Infection

An overnight culture of SC5314 was incubated at 37 °C in a shaker (150 rpm). Cells were washed twice with 50 ml PBS, counted using a hemocytometer, then diluted with PBS to obtain a cell concentration of 1 × 10^6^ cells/ml. CSP was dissolved in sterile MilliQ water to a concentration of 10 mg/ml, and diluted with PBS for intraperitoneal administration to a concentration of 0.01 mg/ml. One day after intravenous infection of *C. albicans* using an infectious dose of 1 × 10^5^ cells in 100 µl, 6–8 weeks old C57BL6/J male mice were treated daily for 7 days with a CSP dose of 0.063 mg/kg body weight. Control mice not on CSP treatment were administered 100 µl of PBS instead. Animals were monitored daily for weight loss and mortality. Animal experiments were conducted according to the rules and guidelines of the Agri-Food and Veterinary Authority (AVA) and the National Advisory Committee for Laboratory Animal Research (NACLAR), Singapore. The experiments were reviewed and approved by the Institutional Review Board of the Biological Resource Center, Singapore (IACUC protocol 140955).

### Isolating CSP Survivors

SC5314 was streaked on YPD plate from −80 °C freezer. The plate was incubated at 37 °C overnight. Several colonies were randomly chosen and suspended in distilled water. 100 µl of the cell suspension (∼1 × 10^7^ cells/ml) were plated on YPD plates supplemented with 100 ng/ml of CSP and incubated at 37 °C for 3 days. Sixty survivors derived from 100 ng/ml of CSP were randomly picked and streaked on YPD plates. The plates were incubated at 37 °C for 36 h. When small and large colonies from each survivor appeared, one small colony and one large colony, were randomly streaked on YPD plates, and incubated at 37 °C for an additional 36 h. Then several small colonies and large colonies were picked and frozen for future experiments.

### Drug Survival Assay

Strains were grown on YPD plates as described above. Several colonies were picked and suspended in distilled water, and cell density was adjusted to 2 × 10^3^ cells/ml. 100 μl of cell suspension were then plated on YPD with or without 100 ng/ml CSP. Plates were incubated at 37 °C for 48 h. Survival percentage was calculated as the ratio of number of colonies on drug plates to the total number of colonies on YPD plates.

### Serial Dilution Spot Assays

Serial dilutions of cell suspension starting from OD 2 were spotted (3 µl/spot) on YPD plates supplemented with the concentrations of HU or CSP indicated in figure legends and incubated at 37 °C for 48 h. Each condition was tested a minimum of three independent times. Growth data were acquired using a desktop scanner and analyzed using a custom R script (available upon request) for automated spot detection and intensity measurements followed by nonlinear curve fitting across the range of serial dilutions. Growth scores were determined as a function of dilution required to reach 50% of the maximum spot intensity of each strain. Relative growth scores were then calculated by normalizing the dilution value against the dilution value of the WT control strain obtained from a YPD control plate spotted in parallel to the plate containing the tested stress condition.

“Next generation sequencing” was performed as described previously (Yang et al. 2017).

### Data Analysis

Statistical analysis was performed in either Prism 6 (GraphPad Software) or R (R Core Team 2016). All custom scripts are available from the authors upon request.

## Supplementary Material

Supplementary data are available at *Molecular Biology and Evolution* online.

## Supplementary Material

msz104_Supplementary_Data
