# Supplementary material for: Aneuploidy Enables Cross-Adaptation to Unrelated Drugs
Source: Mol Biol Evol. 2019 Apr 27;36(8):1768–82. doi: 10.1093/molbev/msz104 (PMC6657732; doi:10.1093/molbev/msz104)

A

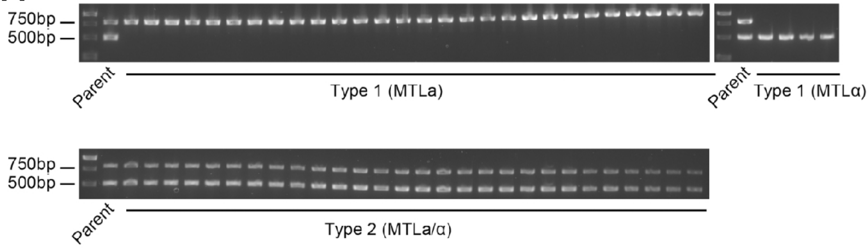
750bp
500bp
Parent
Type 1 (MTLa)
Parent
Type 1 (MTLα)
750bp
500bp
Parent
Type 2 (MTLa/α)

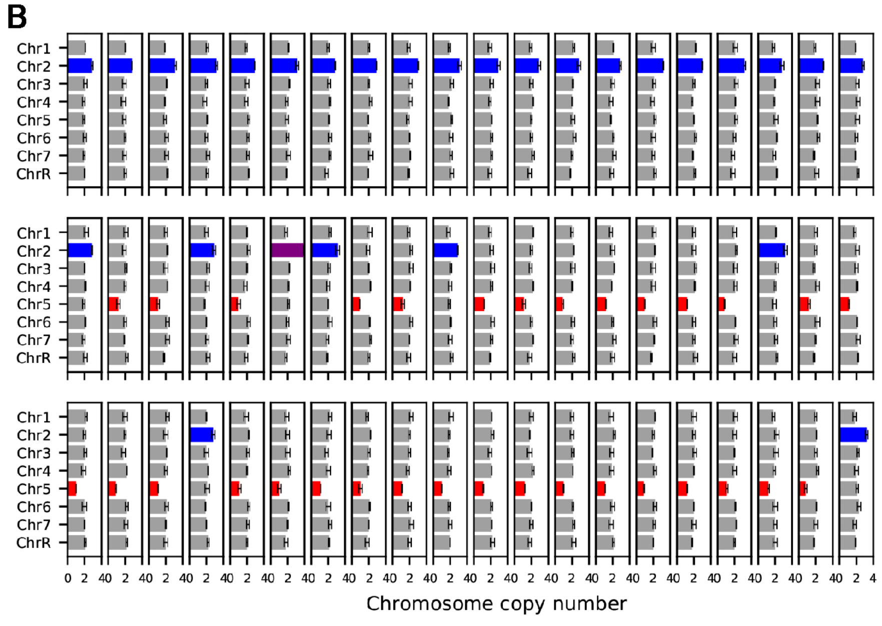
B
Chr1
Chr2
Chr3
Chr4
Chr5
Chr6
Chr7
ChrR
0 2 4
Chromosome copy number

C

Comparison of *GSC1* hot spot region 1 between strains

```
          1900      1910      1920      1930      1940      1950      1960      1970      1980      1990      2000
CaGSC1    AAATTGGTTGAATCTTATTTCTTCTTGACATTGTCTTTAAGAGATCCTATTAGAAACTTGTCGACCATGACAATGAGATGTGTTGGTGAAGTTTGGTACAA
Parent    AAATTGGTTGAATCTTATTTCTTCTTGACATTGTCTTTAAGAGATCCTATTAGAAACTTGTCGACCATGACAATGAGATGTGTTGGTGAAGTTTGGTACAA
FY376     AAATTGGTTGAATCTTATTTCTTCTTGACATTGTCTTTAAGAGATCCTATTAGAAACTTGTCGACCATGACAATGAGATGTGTTGGTGAAGTTTGGTACAA
FY382     AAATTGGTTGAATCTTATTTCTTCTTGACATTGTCTTTAAGAGATCCTATTAGAAACTTGTCGACCATGACAATGAGATGTGTTGGTGAAGTTTGGTACAA
Consensus AAATTGGTTGAATCTTATTTCTTCTTGACATTGTCTTTAAGAGATCCTATTAGAAACTTGTCGACCATGACAATGAGATGTGTTGGTGAAGTTTGGTACAA
```

Comparison of *GSC1* hot spot region 2 between strains

```
          4050      4060      4070      4080      4090      4100      4110      4120      4130      4140      4150
CaGSC1    CAATATTGCTCCTGCCGTTGATTGGATTAGACGTTATACTTTGTCTATTTTCATTGTTTTCTTCATTTCTTTCATTCCATTGGTTGTACAAGAATTGATTG
Parent    CAATATTGCTCCTGCCGTTGATTGGATTAGACGTTATACTTTGTCTATTTTCATTGTTTTCTTCATTTCTTTCATTCCATTGGTTGTACAAGAATTGATTG
FY376     CAATATTGCTCCTGCCGTTGATTGGATTAGACGTTATACTTTGTCTATTTTCATTGTTTTCTTCATTTCTTTCATTCCATTGGTTGTACAAGAATTGATTG
FY382     CAATATTGCTCCTGCCGTTGATTGGATTAGACGTTATACTTTGTCTATTTTCATTGTTTTCTTCATTTCTTTCATTCCATTGGTTGTACAAGAATTGATTG
Consensus CAATATTGCTCCTGCCGTTGATTGGATTAGACGTTATACTTTGTCTATTTTCATTGTTTTCTTCATTTCTTTCATTCCATTGGTTGTACAAGAATTGATTG
```

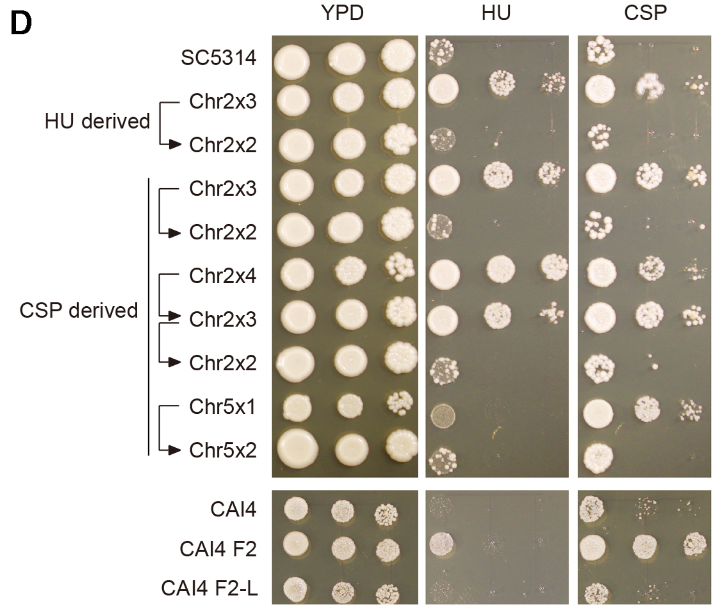
D
YPD
HU
CSP
SC5314
HU derived
Chr2x3
Chr2x2
CSP derived
Chr2x3
Chr2x2
Chr2x4
Chr2x3
Chr2x2
Chr5x1
Chr5x2
CAI4
CAI4 F2
CAI4 F2-L

E

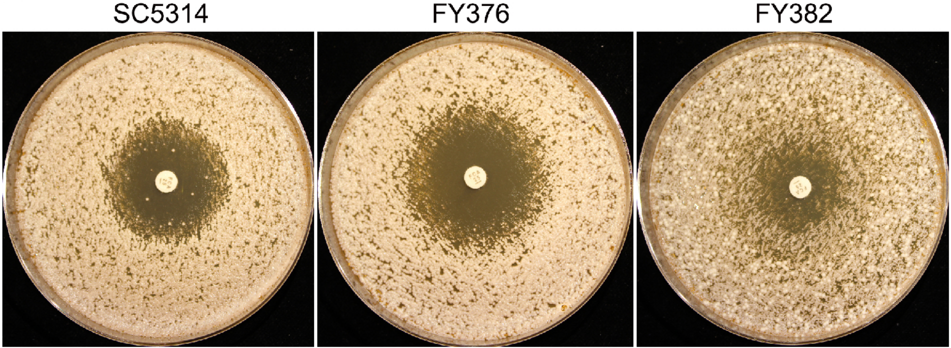

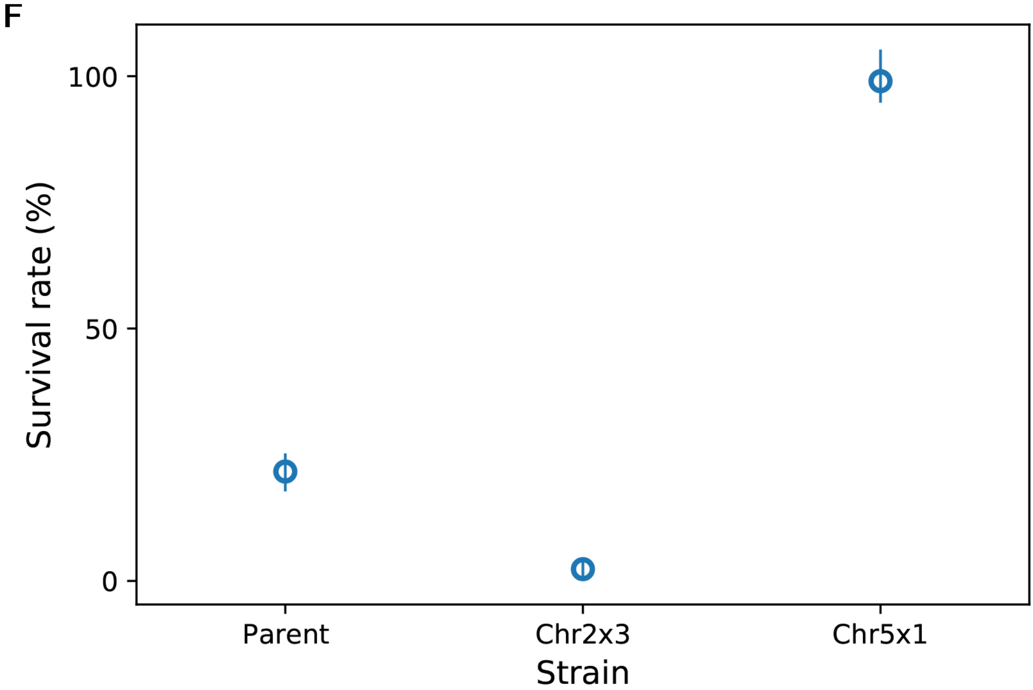
F
100
50
0
Survival rate (%)
Parent
Chr2x3
Chr5x1
Strain

G

RNRs are required for Chr2x3 formation in HU but not in CSP

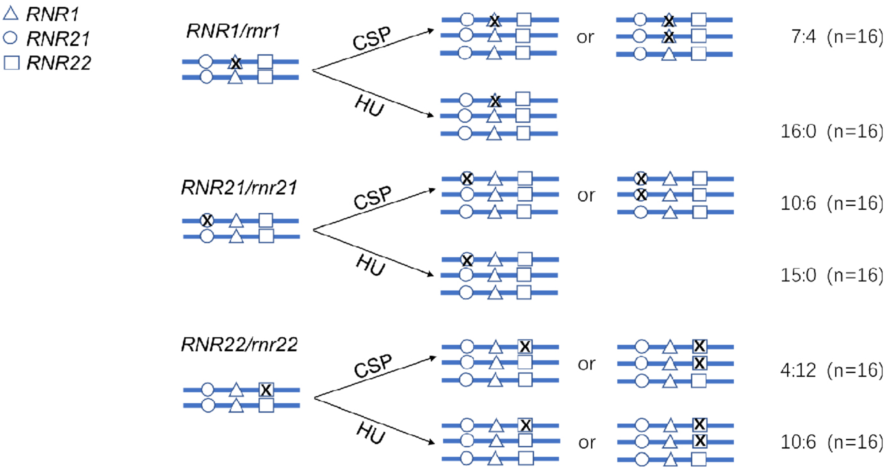

Supplement: msz104_Supplementary_Data [file msz104_supplementary_data.zip › Fig S2ABCDEFG.pdf]
