# Supplementary material for: Aneuploidy Enables Cross-Adaptation to Unrelated Drugs
Source: Mol Biol Evol. 2019 Apr 27;36(8):1768–82. doi: 10.1093/molbev/msz104 (PMC6657732; doi:10.1093/molbev/msz104)
Supplement: msz104_Supplementary_Data [file msz104_supplementary_data.zip › Supplementary text_merged.docx]

Supplementary Material for

Aneuploidy enables cross-adaptation to unrelated drugs

Feng Yang^1,^*, Flora Teoh^2,^*, Alrina Shin Min Tan^2^, Yongbing Cao^3,4^, Norman Pavelka^2,@^, Judith Berman^1,@^

^1^Department of Molecular Microbiology and Biotechnology, School of Molecular Cell Biology and Biotechnology, The George S. Wise Faculty of Life Sciences, Tel Aviv University Tel Aviv, Israel 69978

^2^Singapore Immunology Network (SIgN), Agency for Science, Technology and Research (A*STAR), 8A Biomedical Grove, Immunos #04, Singapore 138648

^3^Department of Vascular Disease, Shanghai TCM-Integrated Hospital, Shanghai University of Traditional Chinese Medicine, Shanghai, China

^4^Shanghai TCM-Integrated Institute of Vascular Disease, Shanghai, China

*These authors contributed equally

^@^Correspondence: [normanpavelka@gmail.com](mailto:normanpavelka@gmail.com) (N.P.) and [jberman@post.tau.ac.il](mailto:jberman@post.tau.ac.il) (J.B.)

# Supplementary Materials and Methods

## Karyotyping of *C. albicans* by quantitative PCR (qPCR)

DNA from frozen cell pellets was extracted using the following method: cells were resuspended in PBS and washed twice with PBS. A digestion buffer containing 1 μl of 0.1 M dithiothreitol (Invitrogen) and 13 μl of 10 mg/mL zymolyase (US Biologicals) in 30 μl PBS was added to cells and incubated at 37 °C for at least 1 h. Cells were then lysed at 95 °C for 5 min and spun down at 3,500 rpm for 5 min. The supernatant was stored at −80 °C. Karyotyping was performed using a qPCR-based method previously developed for *S. cerevisiae* (Pavelka, et al. 2010) and adapted for *C. albicans* (Pavelka et al., manuscript in preparation).

## Colony PCR of the *MTL* locus

PCR reactions were performed in a final volume of 10 µL with 5 µL of 2🞨 Phire Plant Direct PCR Master Mix (Thermo Scientific), 10 µM of forward and reverse primers of both *MTLa1* and *MTLα1* genes (see Table S2 for primer sequences). For each strain, one colony was randomly picked and resuspended in the reaction solution. PCR conditions were: initial denaturation at 94 °C for 3 min, 30 cycles each of denaturation at 94 °C for 30 s, annealing at 62 °C for 30 s, and extension at 72 °C for 1 min, and a final extension at 72 °C for 5 min. 5 µL of PCR product was run in a 1% agarose gel. Gels were stained with ethidium bromide and photographed.

## Sequencing of *GSC1* hotspot mutations

Hot spot regions 1 and 2 of *GSC1* were PCR-amplified and sequenced using primers listed in Table S2. Sequences of the amplified segments were compared to the most current version of the published SC5314 genome sequence (Assembly 22) using BLAST (Altschul, et al. 1997).

# Supplementary Figure Legends

## Fig. S1. Responses of *C. albicans* to anti-cancer and antifungal drugs.

(A-C), Dose-response curves for *C. albicans* in response to acute exposure of doxorubicin, cyclophosphamide and paclitaxel. OD_600_ readings were taken hourly for 6 hours. Growth rates were obtained by calculating the slope of all log_2_OD_600_ readings over time (in hours). (D-H), Karyotypes of representative strains determined by quantitative PCR (qPCR). qPCR assays (Pavelka, et al. 2010) were designed with primers to non-coding regions proximal to the centromere and the telomere on each *C. albicans* chromosome arm. All PCR reactions were performed in technical triplicates. Chromosome copy numbers were determined using the 2^-∆∆Ct^ method (Livak KJ and Schmittgen TD, 2001). Colonies tested were derived from 6 hours of acute exposure to chemotherapeutic drugs HU, cisplatin, MTX at concentrations indicated in the figure. For HU treated survivors, colonies were derived from three independent experiments (line 1, line 2, line 3 as indicated), and colonies of different sizes on YPD+HU plates (described in Materials and Methods) were tested. Trisomy of Chr2 and monosomy of Chr5 were the most common aneuploidies observed. Of 30 colonies isolated after IC_50_ treatment, two had trisomy of Chr2 and two had monosomy of Chr5; of the 30 colonies from IC_90_ treatment, one had trisomy of Chr2 and three had monosomy of Chr5. (I), Caspofungin does not kill all *C. albicans* cells. Isolates representing different clades of *C. albicans* were tested. In E-test assays (Pfaller et al. 2001), approximately 10^5^ cells were plated on YPD plates. In drug survival assays, approximately 10^6^ cells were plated on YPD plates supplemented with drugs at concentrations as indicated in the figure. The plates were incubated at 37˚C for 3 days then photographed.

## Fig. S2. Survivors of caspofungin are aneuploid

(A), Polymerase chain reaction (PCR) amplification of *C. albicans MTL* locus. All sixty survivors derived from SC5314 after exposure to CSP were tested. For each PCR reaction, primers targeting genes *MTLa1* and *MTLα1* were mixed together. (B), Karyotypes of caspofungin survivors determined by quantitative PCR (qPCR), performed as described above (Fig. S1). Karyotypes are displayed vertically with chromosome copy number on the X-axis. Chr2x3 is highlighted in blue; Chr5x1 is highlighted in red; Chr2x4 is highlighted in purple. (C), Sanger sequencing of *GSC1* hot spot regions. Hot spot regions 1 and 2 were amplified in the parent strain SC5314, in one Chr2x3 strain (FY376) and in one Chr5x1 strain (FY382). The PCR amplicons were aligned to the reference sequence of *GSC1* retrieved from Candida Genome Database (CGD) and no mutations were detected. (D-F), Effects of aneuploidies on drug responses. D, Spot assay analysis of susceptibility to HU and CSP of representative strains. 10-fold serial dilutions of cell suspension were spotted (3µl/spot) on YPD plates supplemented with HU (IC50; 2.814 mg/ml) or CSP (50ng/ml) and incubated at 37˚C for 48h. Note that Chr2X3, but not Chr5x1 enables growth on both HU and CSP. E, disc diffusion assay of representative strains. Analyzed strains were: parent, Sc5314; Chr2x3 (FY376), and Chr5x1 (FY382). Approximately 1x10^5^ cells were plated on YPD plates. Plates with FLC disks (25 µg) were incubated at 30˚C for 48 h, and photographed. F, Assay of Survival on FLC plates. Approximately 300 cells per strain (as in Fig S5B) were spread on YPD plates with or without FLC (2 µg/ml). Survival rate (mean of three technical replicates) were determined following incubation at 30˚C for 48 h. G, Role of *RNR* genes in Chr2x3 formation. Approximately 10^6^ cells of heterozygous deletion strains *RNR1/rnr1, RNR21/rnr21, RNR22/rnr22* were plated on YPD plates supplemented with HU (2.814mg/ml) and CSP (100 ng/ml). From each plate, sixteen colonies were randomly tested for Chr2 copy number and *RNR* genes copy number.

## Fig. S3.

(A-C) Weight loss in mice following systemic infection with wild-type or HU-evolved *C. albicans* harboring either ChrRx3 or Chr2x3, with or without CSP treatment. Weight was monitored daily for up to 2 weeks post-infection. Weight change was normalized to weight on Day 0, before infection. Error bars indicate standard deviation. D, Spot assay analysis of susceptibility to HU for strains used in the mice infection experiments. The Chr2x3 strain and the ChrRx3 strain were obtained after 14 days of evolution in HU. 10-fold serial dilutions of cell suspensions were spotted (3µl/spot) onto plates of YPD or YPD supplemented with HU (IC50; 2.814 mg/ml). The plates were incubated at 37˚C for 48h and then photographed.

# Supplementary Tables

## Table S1. Strains used in this study

| **Strain name** | **Relevant genotype** | **Karyotype** | **Description** | **Parental strain** | **Reference / Source** |
| --- | --- | --- | --- | --- | --- |
| SC5314 | Wild type | 2N | Wild-type clinical isolate of *C. albicans* | N/A | (Gillum, et al. 1984) |
| YFT1 | Wild type | 2N, +2 | Chr2x3 strain derived from 6 hours’ treatment with HU (IC_50_) | SC5314 | This study |
| YFT2 | Wild type | 2N | Chr2x2 strain derived from YFT1 | YFT1 | This study |
| YFT3 | Wild type | 2N, +R | ChrRx3 strain | SC5314 | This study |
| FY382 | Wild type | 2N, −5 | Chr5x1 strain derived from exposure to CSP | SC5314 | This study |
| FY383 | Wild type | 2N | Chr5x2 strain derived from YFT382 | FY382 | This study |
| FY376 | Wild type | 2N,+2 | Chr2x3 strain derived from exposure to CSP | SC5314 | This study |
| FY377 | Wild type | 2N | Chr2x2 strain derived from YFT376 | FY376 | This study |
| FY389 | Wild type | 2N, +2, +2 | Chr2x4 strain derived from exposure to CSP | SC5314 | This study |
| FY390 | Wild type | 2N, +2 | Chr2x3 strain derived from YFT389 | FY389 | This study |
| FY391 | Wild type | 2N | Chr2x2 strain derived from YFT390 | FY390 | This study |
| CAI4 | ura3::imm434/ura3::imm434 iro1/iro1::imm434 | 2N | Isogenic to the SC5314 strain. Uridine auxotroph constructed by deletion of the second copy of *URA3* | CAF2-1 | (Selmecki, et al. 2005) |
| CAI4-F2 | Wild type | 2N, +2 | Chr2x3 strain derived from spontaneous genome change | CAI4 | (Selmecki, et al. 2005) |
| CAI4-F2-L | Wild type | 2N | Chr2x2 strain derived from CAI4-F2 | CAI4-F2 | This study |
| YJBT545 | ura3Δ::imm434/ura3Δ::imm434 | 2N | CAI4-2 | CAF2-1 | (Karababa, et al. 2006) |
| YJBT911 | *cna1::hisG/cna1∆::hisG* | 2N | Homogyzous deletion of CNA1 | YJBT545 | (Karababa, et al. 2006) |
| YJBT938 | *crz1∆::hisG/crz1∆::hisG-URA3-higG* | 2N | Homogyzous deletion of CRZ1 | YJBT545 | (Karababa, et al. 2006) |
| YJBT736 | *leu2Δ/leu2Δ his1Δ/his1Δ arg4Δ/arg4Δ URA3/ura3Δ::imm434 IRO1/iro1Δ::imm434* | 2N | SN152 |  | (Vincent, et al. 2013) |
| YJBT744 | *cnb1∆::C.d.HIS1/cnb1∆::C.m.LEU2* | 2N | Homogyzous deletion of CNB1 | SN152 | (Vincent, et al. 2013) |
| FY30 | *RNR1/rnr1::NAT1 flp* | 2N | Heterozygous deletion of *RNR1* | SC5314 | This study |
| FY31 | *RNR21/rnr21::NAT1 flip* | 2N | Heterozygous deletion of *RNR21* | SC5314 | This study |
| FY32 | *RNR22/rnr22::NAT1 flip* | 2N | Heterozygous deletion of *RNR22* | SC5314 | This study |
| FY33 | *RNR1/rnr1::FRT* | 2N | Heterozygous deletion of *RNR1* with nourseothricin marker evicted | FY30 | This study |
| FY34 | *RNR1/rnr1::FRT RNR21/rnr21::NAT1 flp* | 2N | Heterozygous deletion of *RNR1* and *RNR21* | FY33 | This study |
| FY35 | *RNR1/rnr1::FRT RNR22/rnr22::NAT1 flp* | 2N | Heterozygous deletion of *RNR1* and *RNR22* | FY33 | This study |
| FY36 | *RNR1/RNR1/rnr1::NAT1 flp* | 2N, +2 | Chr2x3 strain obtained by exposing FY30 to HU | FY30 | This study |
| FY37 | *RNR1/RNR1//rnr1::NAT1 flp* | 2N, +2 | Chr2x3 strain obtained by exposing FY30 to CSP | FY30 | This study |
| FY38 | *RNR1/rnr1::NAT1 flp /rnr1::NAT1 flp* | 2N, +2 | Chr2x3 strain obtained by exposing FY30 to CSP | FY30 | This study |
| FY39 | *RNR21/RNR21/rnr21::NAT1 flp* | 2N, +2 | Chr2x3 strain obtained by exposing FY31 to HU | FY31 | This study |
| FY40 | *RNR21/RNR21//rnr21::NAT1 flp* | 2N, +2 | Chr2x3 strain obtained by exposing FY31 to CSP | FY31 | This study |
| FY41 | *RNR21/rnr21::NAT1 flp /rnr21::NAT1 flp* | 2N, +2 | Chr2x3 strain obtained by exposing FY31 to CSP | FY31 | This study |
| FY42 | *RNR22/RNR22/rnr22::NAT1 flp* | 2N, +2 | Chr2x3 strain obtained by exposing FY32 to HU | FY32 | This study |
| FY43 | *RNR22/rnr22::NAT1 flp /rnr22::NAT1 flp* | 2N, +2 | Chr2x3 strain obtained by exposing FY32 to CSP | FY32 | This study |
| FY44 | *RNR22/RNR22/rnr22::NAT1 flp* | 2N, +2 | Chr2x3 strain obtained by exposing FY32 to HU | FY32 | This study |
| FY45 | *RNR22/rnr22::NAT1 flp /rnr22::NAT1 flp* | 2N, +2 | Chr2x3 strain obtained by exposing FY32 to CSP | FY32 | This study |
| FY46 | *RNR1/RNR1//rnr1::NAT1 flp* | 2N, +2 | Heterozygous deletion of *RNR1* | YFT1 | This study |
| FY47 | *RNR21/RNR21//rnr21::NAT1 flp* | 2N, +2 | Heterozygous deletion of *RNR21* | YFT1 | This study |
| FY48 | *RNR22/RNR22//rnr22::NAT1 flp* | 2N, +2 | Heterozygous deletion of *RNR22* | YFT1 | This study |
| FY48 | *RNR1/RNR1/rnr1::FRT* | 2N, +2 | Heterozygous deletion of *RNR1* with nourseothricin marker evicted | FY46 |  |
| FY49 | *RNR1/rnr1::FRT/rnr1::NAT1 flp* | 2N, +2 | Deletion of two alleles of *RNR1* | FY48 |  |
| FY50 | *RNR1/RNR1/rnr1::FRT RNR21/RNR21//rnr21::NAT1 flp* | 2N, +2 | Heterozygous deletion of *RNR1* and *RNR21* | FY48 |  |
| FY51 | *RNR1/RNR1/rnr1::FRT RNR22/RNR22//rnr22::NAT1 flp* | 2N, +2 | Heterozygous deletion of *RNR1* and *RNR22* | FY48 |  |

## Table S2. Primers used in this study

| **Primer name** | **Sequence (5’ 🡪 3’)** | **Purpose** | **Reference / Source** |
| --- | --- | --- | --- |
| MTLa1-F | TTGAAGCGTGAGAGGCAGGAG | *MTL* locus genotyping | (Panwar, et al. 2003) |
| MTLa1-R | GTTTGGGTTCCTTCTTTCTCATTC | *MTL* locus genotyping | (Panwar, et al. 2003) |
| MTLα1-F | TTCGAGTACATTCTGGTCGCG | *MTL* locus genotyping | (Panwar, et al. 2003) |
| MTLα1-R | TGTAAACATCCTCAATTGTACCCGA | *MTL* locus genotyping | (Panwar, et al. 2003) |
| GSC1 HS1-F | CGGTGCTCAACATTTGAGTCGTCGTAT | *GCS1* PCR (hotspot region 1) | (Yang, et al. 2013) |
| GSC1-HS1-R | TTGATTTCCATTTCCGTGGTAGCTAAA | *GCS1* PCR (hotspot region 1) | (Yang, et al. 2013) |
| GSC1-HS1-Seq | CGGCATATGCTGTGTCGATTGT | *GCS1* sequencing (hotspot region 1) | (Yang, et al. 2013) |
| GSC1-HS2-F | TGCTGGTATGAATGCCATGATGAGAGG | *GCS1* PCR (hotspot region 2) | (Yang, et al. 2013) |
| GSC1-HS2-R | GGTGCTTGCCAATGAGAAACTGTACC | *GCS1* PCR (hotspot region 2) | (Yang, et al. 2013) |
| GSC1-HS2-Seq | TTGGTGCTGGTATGGGAGAACA | *GCS1* sequencing (hotspot region 2) | (Yang, et al. 2013) |
| CaRNR1-US-F | GAAACTAAACGATAGGTGAACCA | *RNR1* deletion | This study |
| NAT1-CaRNR1-US-R | GTATAGGAACTTCCTCGAGGGGCGACACGTAAAAAATATAGCG | *RNR1* deletion | This study |
| NAT1-CaRNR1-DS-F | AGATCCACTAGTTCTAGAGCGGGTTCTTTTCTGAGCGATGTTG | *RNR1* deletion | This study |
| CaRNR1-DS-R | GCAGAACCCTGAAATCAAATG | *RNR1* deletion | This study |
| CaRNR1-USD-F | CAGATGTTATTTGAACGACGG | *RNR1* deletion (diagnostic PCR) | This study |
| CaRNR1-DSD-R | AGTATGAAACGGTTGCAACC | *RNR1* deletion (diagnostic PCR) | This study |
| CaRNR21-US-F | ACGCGTAGTGGGAAAATG | *RNR21* deletion | This study |
| NAT1-CaRNR21-US-R | GTATAGGAACTTCCTCGAGGGCTTGAAATAATTTGGAAGAGGG | *RNR21* deletion | This study |
| NAT1-CaRNR21-DS-F | AGATCCACTAGTTCTAGAGCGGTGATTTCAGTTCGTTTTCCC | *RNR21* deletion | This study |
| CaRNR21-DS-R | CGAAATCTCTTGTCTTCCTTTAC | *RNR21* deletion | This study |
| CaRNR21-USD-F | TTCAAAATATCCAAGATCGGG | *RNR21* deletion (diagnostic PCR) | This study |
| CaRNR21-DSD-R | GCAGGAAAGGAAGTTGACG | *RNR21* deletion (diagnostic PCR) | This study |
| CaRNR22-US-F | CCAGACTTGATTTTGCGC | *RNR22* deletion | This study |
| NAT1-CaRNR22-US-R | GTATAGGAACTTCCTCGAGGGTAGTTAAGTGGAATTTTTGCGC | *RNR22* deletion | This study |
| NAT1-CaRNR22-DS-F | AGATCCACTAGTTCTAGAGCGGGGACTTTGAAAACGGGC | *RNR22* deletion | This study |
| CaRNR22-DS-R | CTCGGTACCATGGGAAGAG | *RNR22* deletion | This study |
| CaRNR22-USD-F | GACTTTGGTTAATTTGATCCG | *RNR22* deletion (diagnostic PCR) | This study |
| CaRNR22-DSD-R | CAACGTTTAAGAACAAAAGTACCC | *RNR22* deletion (diagnostic PCR) | This study |

# Supplementary References

Altschul SF, Madden TL, Schaffer AA, Zhang J, Zhang Z, Miller W, Lipman DJ. 1997. Gapped BLAST and PSI-BLAST: a new generation of protein database search programs. Nucleic Acids Res 25:3389-3402.

Gillum AM, Tsay EY, Kirsch DR. 1984. Isolation of the Candida albicans gene for orotidine-5'-phosphate decarboxylase by complementation of S. cerevisiae ura3 and E. coli pyrF mutations. Mol Gen Genet 198:179-182.

Karababa M, Valentino E, Pardini G, Coste AT, Bille J, Sanglard D. 2006. CRZ1, a target of the. calcineurin pathway in Candida albicans. Mol Microbiol 59:1429-1451.

Livak KJ, Schmittgen TD. 2001. Analysis of relative gene expression data using real-time quantitative PCR and the 2(^-∆∆CT^) method. *Methods*. 25(4):402-408.

Panwar SL, Legrand M, Dignard D, Whiteway M, Magee PT. 2003. MFalpha1, the gene encoding. the alpha mating pheromone of Candida albicans. Eukaryot Cell 2:1350-1360.

Pavelka N, Rancati G, Zhu J, Bradford WD, Saraf A, Florens L, Sanderson BW, Hattem GL, Li R. 2010. Aneuploidy confers quantitative proteome changes and phenotypic variation in budding yeast. Nature 468:321-325.

Pfaller MA, Messer SA, Mills K, Bolmstrom A, Jones RN. 2001. Evaluation of Etest method for determining caspofungin (MK-0991) susceptibilities of 726 clinical isolates of Candida species. *J Clin Microbiol*. 39(12):4387-4389.

Selmecki A, Bergmann S, Berman J. 2005. Comparative genome hybridization reveals widespread. aneuploidy in Candida albicans laboratory strains. Mol Microbiol 55:1553-1565.

Vincent BM, Lancaster AK, Scherz-Shouval R, Whitesell L, Lindquist S. 2013. Fitness trade-offs. restrict the evolution of resistance to amphotericin B. PLoS Biol 11:e1001692.

Yang F, Kravets A, Bethlendy G, Welle S, Rustchenko E. 2013. Chromosome 5 monosomy of. Candida albicans controls susceptibility to various toxic agents, including major antifungals. Antimicrob Agents Chemother 57:5026-5036.
