# Supplementary figures and images for: Aneuploidy Enables Cross-Adaptation to Unrelated Drugs
Source: Mol Biol Evol. 2019 Apr 27;36(8):1768–82. doi: 10.1093/molbev/msz104 (PMC6657732; doi:10.1093/molbev/msz104)

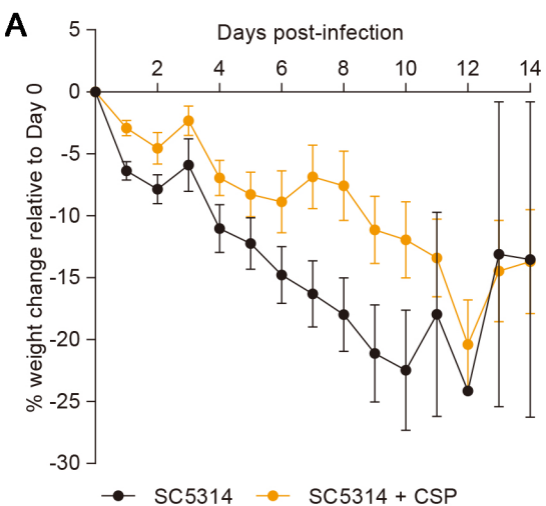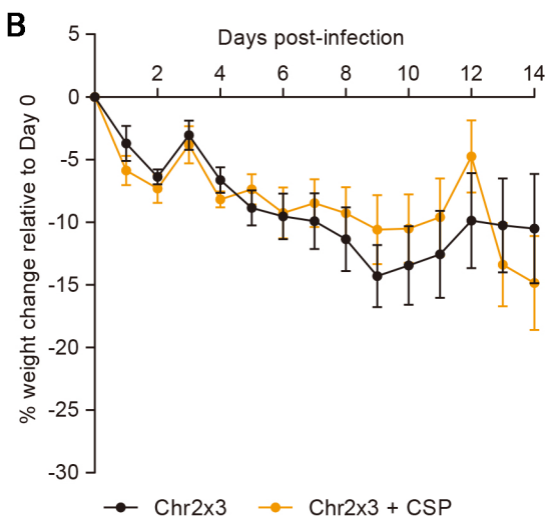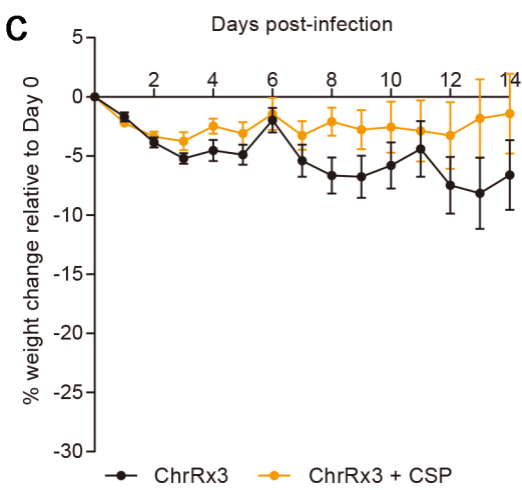

D

YPD

HU

Parent

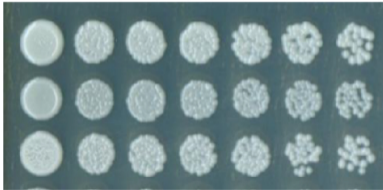

ChrRx3

Chr2X3

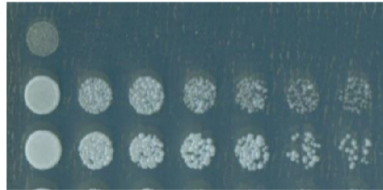

Supplement: msz104_Supplementary_Data [file msz104_supplementary_data.zip › Fig S3ABCD.pdf]
